# Enabling in vivo imaging in low‐resource settings: Computed tomography imaging of gold‐loaded polymersomes for the detection of glioblastoma

**DOI:** 10.1002/btm2.70109

**Published:** 2026-01-27

**Authors:** Emily Barnett, Joey Lavalla, Pranavi Thatavarthi, Isabel Ray, Taylor Hamas, Jessica Jager, Vaishnavi Kanduri, Jasmine White, Elizabeth Singleton, Jordan Drinks, Megan Pitz, Angela Alexander‐Bryant, Jessica Larsen

**Affiliations:** ^1^ Department of Chemical and Biomolecular Engineering Clemson University Clemson South Carolina USA

**Keywords:** computed tomography, glioblastoma, gold nanoparticles, polymersomes

## Abstract

Glioblastoma (GBM) is one of the most aggressive and rapidly progressing brain tumors, characterized by a low survival rate, in part due to insufficient diagnostic tools. Computed tomography (CT), although widely available, is limited in use for GBM diagnosis by the suboptimal performance of current clinically approved contrast agents. This study focuses on the development of gold nanoparticle (AuNP)‐loaded polymersomes (AuPs) to improve the detection of GBM. We synthesized polyethylene glycol‐b‐polylactic acid (PEG‐b‐PLA) polymersomes with high AuNP loading. Increasing the concentrations of AuNPs in polymersomes resulted in enhanced contrast using clinical CT. Furthermore, AuPs bound to cell‐penetrating peptide TAT were cytocompatible with U87‐MG GBM cells at concentrations up to 100 mg/mL. Uptake studies using both fluorescence microscopy and flow cytometry confirmed the internalization of AuPs into GBM cells, with a direct correlation between AuP concentration and uptake efficiency. MicroCT imaging also confirmed a similar trend; >300% enhanced contrast compared to PBS controls was observed with increasing concentrations of AuPs and was maintained in vivo at 337–863 HU. Overall, these results demonstrate that a polymersome‐based system for AuNPs enhances CT image contrast, suggesting that this approach could be feasible for improving GBM detection via CT.

**Translational Impact Statement**

This research develops a novel computed tomography contrast agent that could enhance the usability of CT for tumor imaging. Gold‐loaded polymersomes demonstrate high CT contrast *in vitro* and *in vivo* due to their high electron density, intracellular delivery, and biocompatibility. This alternative to standard contrast agents could increase visualization of tumor sites, allow for timely detection, and enable identification of tumor margins via CT, which is more widely accessible than MRI. Future studies should focus on the direct applicability to in situ cancer models.

## 1 INTRODUCTION

Glioblastoma (GBM) is one of the deadliest cancers, accounting for 50.1% of all primary malignant brain tumors, and is associated with an abysmal 6.9% five‐year survival rate.^1^ This tumor aggressively infiltrates healthy brain tissue through rapidly growing tendrils, facilitating the migration of malignant cells into surrounding tissue.^2^ Due to its rapid progression, GBM is often diagnosed as a grade four tumor, with no indicators to suggest tumor presence early in development.^3^ This delay in diagnosis contributes to poor prognosis and low survival rates among GBM patients. There is evidence that improved outcomes, including expanded overall survival times, are linked to total surgical resections^4^; most recurrent GBM tumors occur at or near the original site, indicating the powerful role of the tumor cells left behind. However, total resections can be challenging to accomplish without high‐quality imaging of the tumor margins as a surgical guide.^5^, ^6^, ^7^

Magnetic resonance imaging (MRI) is currently the most common and informative diagnostic method used for GBM detection. However, high‐field MRI needed for this surgical guidance can be difficult to access and costly for patients. Studies indicate that only 66% of medical institutions have MRI machinery, of which an increasing but still small fraction possess the high‐field equipment required to produce detailed spatiotemporal images of the brain to guide surgical interventions.^8^ It has also been observed that MRI machine access is typically restricted to areas that are middle to high‐income,^9^ decreasing the accessibility in low‐income areas. Furthermore, in the more rural counties with less access to MRI machines for cancer diagnostics, there is also a higher prevalence of behaviors linked to cancer (obesity, smoking, binge drinking) and a decreased prevalence of regular cancer screenings.^10^ The rural and low‐income areas are therefore at greater risk of having their cancer go undiagnosed and untreated. Compounding this issue are the major concerns associated with high MRI costs to patients^11^ which can vary from patient to patient, depending on health insurance.

Computed tomography (CT) imaging is emerging as an alternative for GBM detection, as it is already commonly used to visualize and assess tumors for several reasons. CT imaging manipulates x‐rays captured at different angles to reconstruct cross‐sectional images of targeted areas.^12^ It can provide detailed anatomical cross‐sectional images of the body, which gives a higher level of detail of both the tumor and its surrounding structures.^13^ CT scans are also quick in comparison to MRI, making it more suitable in emergencies and for patients who may have difficulty remaining still for extended periods.^14^ The quicker time frame can also enable real‐time guidance for procedures, which allows for accurate tumor targeting during biopsies or surgeries^15^, ^16^, ^17^, ^18^ and image‐guided drug delivery. Because CT is excellent at distinguishing soft tissues from bone, it can detect any calcifications present within the tumor, which could be critical for surgical resection.^19^ With regards to accessibility, studies in various states clearly identify an increased availability of CT machines in comparison to MRI machines; specifically, a study in Minnesota identifies 39 counties that do not have MRI machines, while there were only 14 counties without CT machines,^20^ indicating increased availability of CT in comparison to MRI. Some studies have found that 96% of all emergency departments are equipped with CT scanners.^8^ CT is also a more cost‐effective diagnostic tool than MRI,^11^ with a reported average annual cost of $2670.24 USD per kilowatt‐hour for a head scan compared to $42,202.96 USD per kilowatt‐hour for a 3 T MRI brain scan.^21^

While CT imaging has many advantages for tumor evaluation, the current gold standard iodinated contrast agents used in CT can cause adverse effects, including a risk of contrast‐induced nephropathy (CIN),^22^ and cannot cross the BBB,^22^ only entering tumors through leaky vasculature, which makes well‐defined regions of GBM difficult to image via CT. Only low doses of these contrast agents are approved by the US Food and Drug Administration (FDA), since large doses and long‐term exposure can lead to thyroid dysfunction, contrast‐induced nephropathy, and severe allergic reactions.^23^ However, imaging GBM effectively for diagnosis and surgical guidance with CT would require higher doses of contrast agents, as image quality is directly related to contrast concentration. Increasing the contrast concentration would increase the radiation dose, enhancing image visibility, but also increasing the associated risks.^24^

As an alternative to standard contrast agents, the use of gold nanoparticles (AuNPs) to increase the contrast in imaging modalities has been explored because of their high electron density and biocompatibility.^25^ The high electron density of AuNPs (19.32 g/cm^3^) compared to that of standard iodine‐based agents (4.9 g/cm^3^) results in enhanced and refined CT images.^26^ However, AuNPs can be extremely small, and their size dictates their *in vivo* fate,^27^ with smaller particles being cleared by the kidneys. It is also important that enough AuNPs accumulate in the tumor site to enable high CT image quality. Polymersomes are a promising option to facilitate on‐target AuNP delivery of a high concentration of contrast agents for CT imaging. Polymersomes are highly tunable vesicles synthesized from amphiphilic block copolymers and are thermodynamically spherical in shape. Through hydrophobic interactions and control over the hydrophilic/hydrophobic ratio, these vesicles consist of a hollow structure with a hydrophilic outer shell, hydrophobic middle layer, and hydrophilic inner layer that allows for diverse cargo loading.^28^ Our previous studies demonstrate that they can be used to facilitate the delivery of metallic nanoparticles as contrast agents.^29^

**FIGURE 1:**
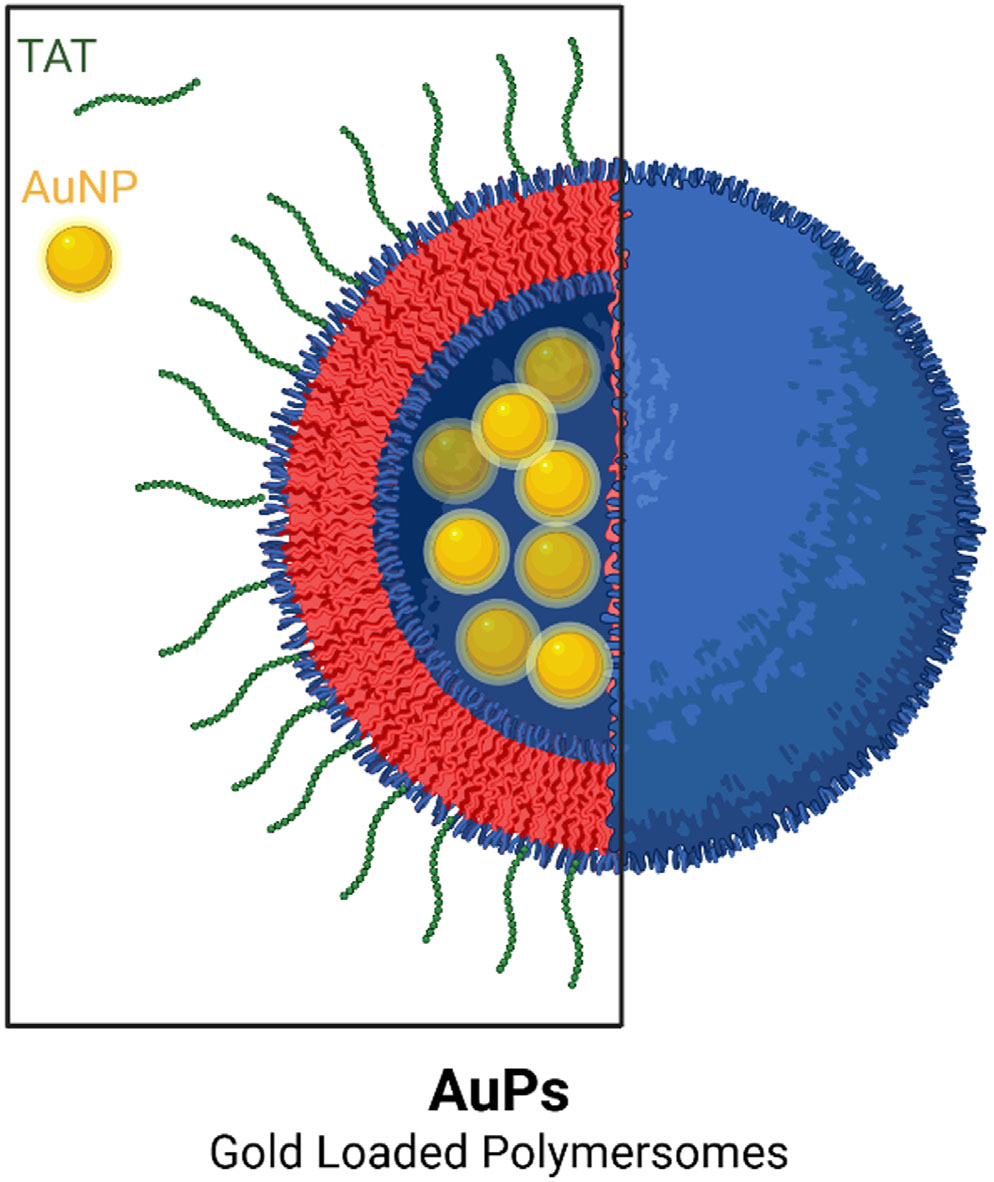
Schematic representation of gold‐loaded polymersomes (AuPs). PEG‐b‐PLA + MAL‐PEG‐b‐PLA polymersomes are loaded with AuNPs and labeled with TAT.

Here, we explore the use of polyethylene glycol (PEG)‐b‐polylactic acid (PLA) polymersomes to facilitate the cellular entry and enhanced CT contrast capabilities of AuNPs. PLA is a biodegradable, biocompatible polyester, and PEG provides hydrophilicity, non‐immunogenicity, and extended circulation times.^30^, ^31^, ^32^ As a technological proof of concept, we use a generic cell‐penetrating peptide TAT on the surface of polymersomes.^22^ TAT attachment was facilitated by the introduction of a 50:50 blend of maleimide (MAL) functionalized PEG‐b‐PLA and the addition of a cysteine on TAT. Future studies will explore the use of targeting ligands to facilitate on‐target delivery, which has been a successful approach to aid polymersome transport in vivo.^33^, ^34^, ^35^, ^36^, ^37^ Specifically, we have shown that PEG‐b‐PLA polymersomes can be trafficked to the peripheral and central nervous system using a peptide of the rabies virus glycoprotein, RVG29R.^33^ We observed that PEG‐b‐PLA polymersomes can encapsulate concentrations of AuNPs around 0.1 mg/mg of polymersomes, facilitate their entry into GBM cells with very limited toxicity, enhance the CT contrast observed via phantom images, and are easily detectable post‐injection in mice. The work presented demonstrates the development of novel CT imaging contrast agents, gold‐loaded polymersomes (AuPs), that could enhance the usability of CT and increase access to high‐quality healthcare (Figure 1).

## 2 RESULTS

### 2.1 Characterization of unlabeled and TAT‐labeled polymersomes

**FIGURE 2:**
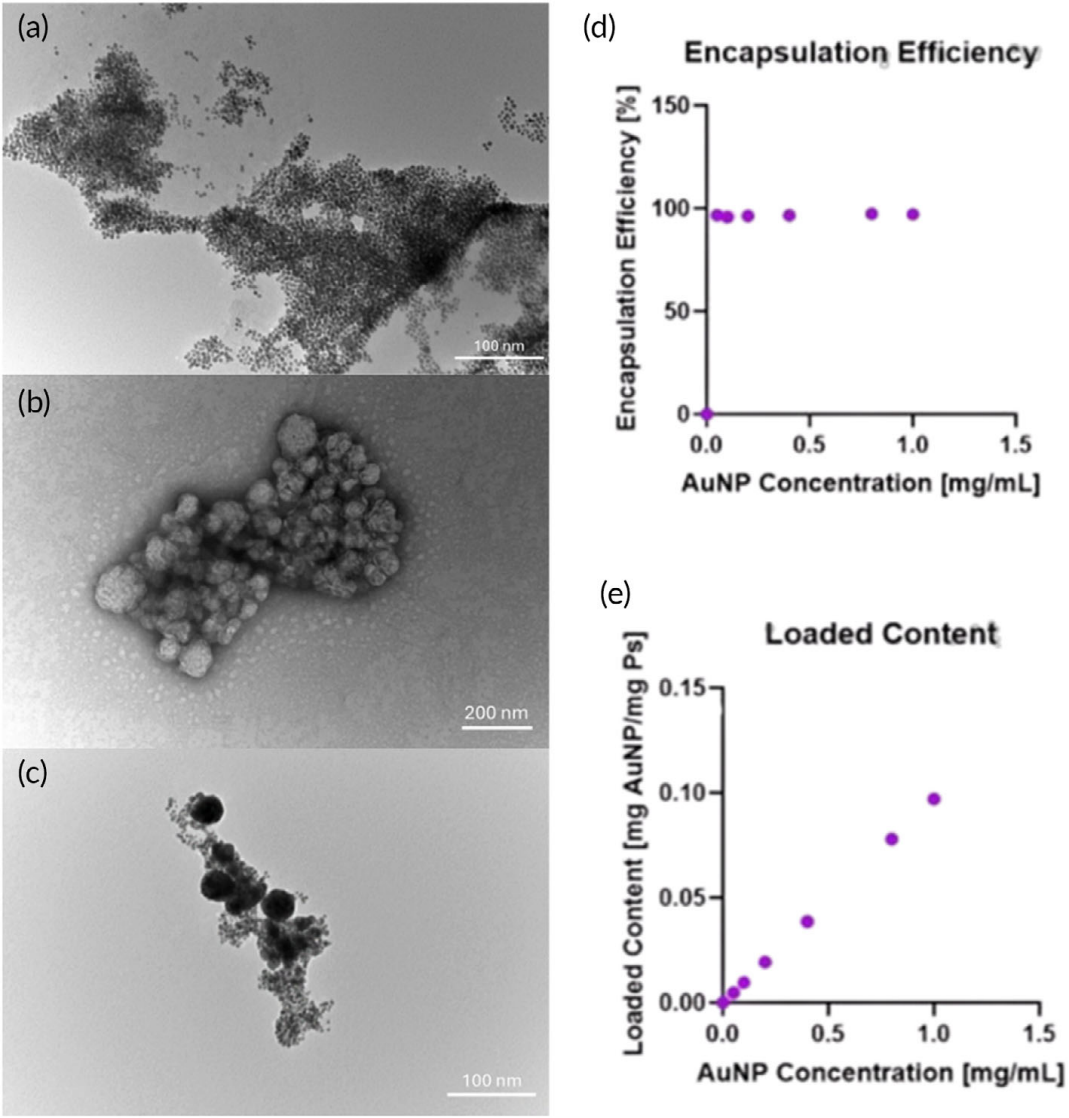
Transmission electron microscopy images of (a) free AuNPs (b) unloaded TAT‐ conjugated polymersomes and (c) filtered AuPs. Absorbance of gold nanoparticles and gold‐loaded polymersomes were measured at a wavelength of 565 nm. Gold was then loaded into the polymersomes in concentrations of 0, 0.05, 0.1, 0.2, 0.4, 0.8, and 1 mg/mL, and the absorbance was measured. (d) The encapsulation efficiency of AuNPs into polymersomes was quantified based on AuNP concentration (*n* = 3). (e) The loaded content of gold into the polymersomes was quantified versus AuNP concentration added (*n* = 3).

Polymersome characterization was performed using dynamic light scattering (DLS) to confirm the diameter, polydispersity index (PDI), and charge (zeta‐potential) of the synthesized particles. The 50:50 PEG‐b‐PLA:MAL‐PEG‐b‐PLA polymersomes had an average size around 114 nm and an average PDI of less than 0.1 (Table 1). After the addition of TAT, the size and PDI remained similar, with an average diameter near 115 nm and a PDI of less than 0.1. Zeta potential measurements revealed an average negative charge near −36 mV for unlabeled 50:50 PEG‐b‐PLA: MAL‐PEG‐b‐PLA polymersomes, whereas TAT‐functionalized polymersomes had an increased zeta potential to a highly positive charge around 26 mV, confirming successful TAT functionalization. For further characterization of the AuNPs, unloaded polymersomes, and AuPs, TEM images were obtained to confirm the size, shape, and distribution of the synthesized nanoparticles. Sterile AuNPs were imaged to confirm their spherical shape and small 5 nm size to ensure their ability to be encapsulated into polymersomes (Figure 2a). TEM images of the unloaded TAT‐functionalized polymersomes and filtered AuPs were obtained (Figure 2b). Images confirmed that the spherical shape of both unloaded and filtered AuPs, as well as their size, was consistent with the DLS data. TEM images visually demonstrated the successful encapsulation of gold AuNPs into the polymersomes through darker AuNP spheres located within the hollow polymersomes to form our AuPs (Figure 2c).

**TABLE 1:** Characteristics of PEG–PLA and MAL‐PEG–PLA polymersomes synthesized in a 50:50 ratio in 2% mannitol‐H_2_O solution before and after TAT functionalization (*n* = 6).

TAT	Diameter (nm)	PDI (−)	Z Potential (mV)
−	113.95 ± 0.08	0.074 ± 0.02	−36.53 ± 2.28
+	114.77 ± 0.94	0.075 ± 0.01	25.94 ± 2.14

To determine maximum encapsulation, polymersomes were loaded with AuNPs at various concentrations (0–1 mg/mL) and evaluated to determine the encapsulation efficiency (%) and loaded content in AuPs. The percentage of Au loaded into the polymersomes was quantified by measuring the absorbance of the samples at 565 nm to determine the amount of unencapsulated Au (Figure 2d). The encapsulation efficiency of all concentrations of Au remained high (>99%) across all concentrations explored. The loaded content of the polymersomes was also evaluated to analyze the ability of the TAT‐functionalized polymersomes to load and deliver the contrast agent. Figure 2e demonstrates that as the gold concentration increased, so did the amount of AuNP loaded into polymersomes, with a maximum calculated loading of 0.097 mg AuNP/mg polymersome. This indicates that we did not identify a maximum loading capacity of AuNPs in AuPs in our study. Therefore, a concentration of 1 mg/mL of AuNPs was selected for the duration of the study due to the highest loaded content, ultimately having the best chance to improve visualization in CT.

### 2.2 Phantom CT images of varying concentrations of AuNPs and AuPs

**FIGURE 3:**
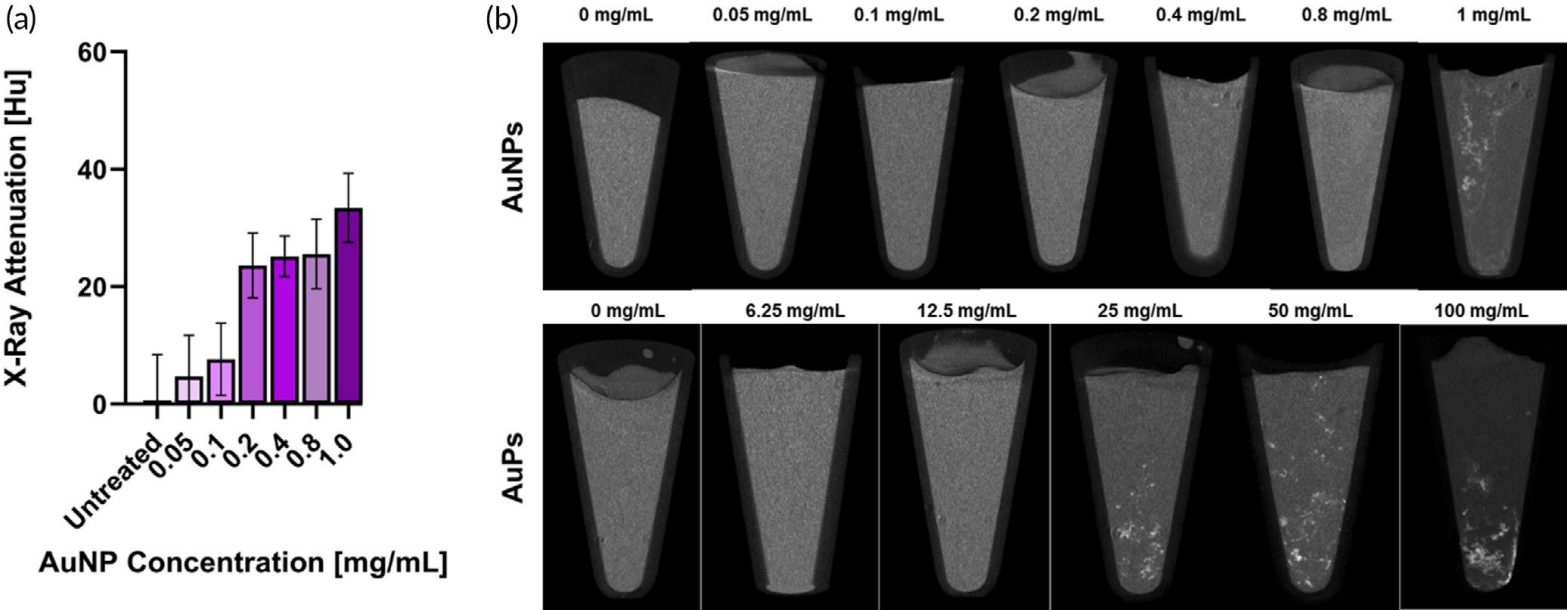
(a) CT image analysis revealed increasing x‐ray attenuation values with increasing concentrations of AuNPs using clinical CT, confirming their value as CT contrast agents (*n* = 3), (b) Micro CT images of AuNPs (top) at concentrations of 0, 0.05, 0.1, 0.2, 0.4, 0.8, and 1 mg/mL and AuPs (bottom) at concentrations of 0, 6.25, 12.5, 25, 50, and 100 mg/mL (*n* = 3; image representative) highlight the differences between contrast enhancement between the two groups. Specifically, AuPs can facilitate large contrast enhancement in microCT that increases with increasing concentration.

To confirm the ability of AuNPs to improve the visual contrast in CT, phantom images taken on an Aquilion TSX‐101A CT scanner (Clinical CT) at increasing concentrations (Figure S1). The results revealed an increase in signal intensity as the concentration of AuNPs increased. The highest concentration of AuNPs (1 mg/mL) resulted in images with the highest intensity, also confirmed via X‐ray attenuation measurements. The lowest and highest concentration samples exhibited X‐ray attenuation values of 0 and 33.44, respectively, which were consistent with the visual observations (Figure 3a). The conclusions drawn from the visual increase in the contrast of AuNPs, as well as the X‐ray attenuation values, aid in the determination of 1 mg/mL AuNPs as the ideal concentration of gold loaded into polymersomes used in the current study.

To follow up, we compared the CT contrast of AuNPs and AuPs at varying concentrations. Polymersomes were loaded with 1 mg/mL AuNPs and imaged up to 100 mg AuPs/mL using a Bruker SkyScan 1176 Micro CT machine. Increasing the volume of polymersomes loaded with a consistent gold concentration aided in increasing the contrast in CT compared to AuNPs alone (Figure 3b). The darkening of the background indicates a more intense signal from the AuPs than the AuNPs. Contrast enhancement was quantified using ImageJ (Figure S2) and demonstrated that the highest enhancement occurred at AuP concentrations between 25 and 100 mg/mL.

### 2.3 In vitro analysis of AuNP and AuPs cytocompatibility

**FIGURE 4:**
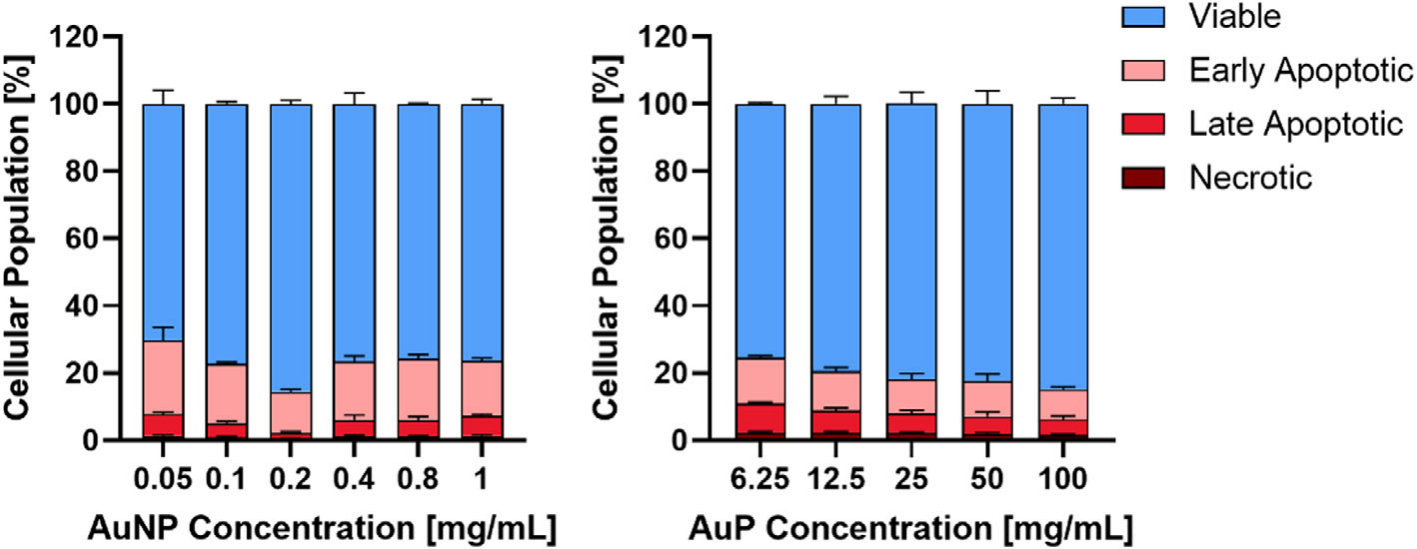
Annexin V/PI viability assay shows minimal toxicity from AuNPs and AuPs. Demonstrating >80% viability at the highest concentration of AuPs, 100 mg/mL, which showed the greatest CT contrast.

The cytotoxicity of AuNPs and AuPs was analyzed *in vitro* using the MTS assay. Figure S3A shows that after 48 h, U87‐MG cells treated with varying concentrations of AuNPs exhibited an increase in viability with increasing concentrations of AuNPs. The 0.8 and 1 mg/mL AuNP concentrations showed 195.04% and 218.20% viability values, respectively. TAT‐labeled polymersomes were cytocompatible up to 100 mg/mL after 48 h, with viability greater than 80% across all concentrations (0–100 mg/mL) (Figure S3B). These results were further validated using an ANOVA analysis of variance, which revealed statistically significant differences in the viability of AuNP concentrations of 0.4, 0.8, and 1 mg/mL, and no statistical difference between the concentrations of AuPs from that of the control group. As a secondary confirmation of cytocompatibility, Annexin V/PI assays were performed (Figures 4 and S4), demonstrating that incubation with both AuNPs and AuPs resulted in a cell population viability of greater than 80%. Incubation with AuPs leads to a greater concentration of viable cells.

### 2.4 Cellular uptake and quantification of AuNPs and AuPs in U87‐MGs

**FIGURE 5:**
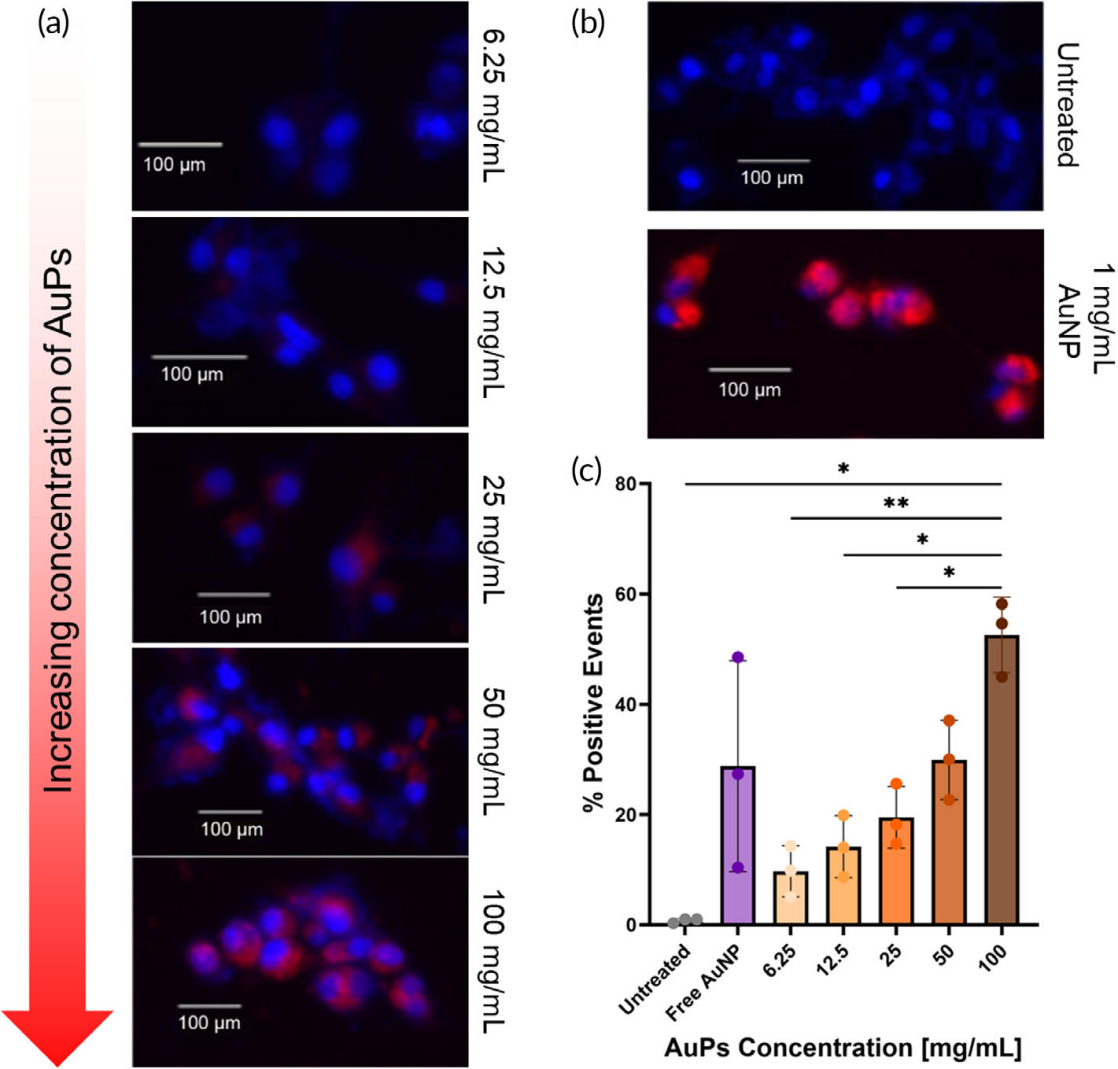
AuPs facilitate increased cellular uptake in U87‐MG Cells. Cells were imaged 24 h post‐treatment with an objective of 20X in PBS. (a) AuPs incubated with U87‐MG cells at increasing concentrations from 6.25 to 100 mg/mL. (b) Untreated, negative control and positive control (1 mg/mL AuNP treated) images. (c) Flow cytometry confirmed uptake of AuNPs and AuPs 24 h post treatment, with increasing AuPs uptake as concentration increases, as observed in Figure 5a. *n* = 3, *p < 0.05, **p < 0.01.

The cellular uptake of AuNPs and AuPs was assessed using fluorescence imaging after 24 h. Cells were treated with varying concentrations of AuPs loaded with fluorescently tagged AuNPs ranging from 6.25 to 100 mg/mL, as well as a free 1 mg/mL AuNP positive control group. U87‐MG cell nuclei were stained using DAPI, and the gold nanoparticles were tagged with a Texas red fluorophore for visualization. Figure 5a,b shows the presence and internalization of both AuNPs and AuPs into cells; the localization of cell nuclei and AuPs suggests cytoplasmic delivery of the treatments. Imaging revealed an increase in the fluorescence as the concentration of AuPs increased (Figure 5a), whereas no fluorescence was observed in the untreated group (Figure 5b). Flow cytometry quantified that after treatment with 100 mg/mL AuPs, ~53% of cells showed AuNP internalization, which was a statistically significant increase compared to the 1 mg/mL dose of AuNPs, which led to ~29% of cells with AuNP internalization (Figures 5c and S5).

### 2.5 In vivo assessment of microCT contrast of AuPs

**FIGURE 6:**
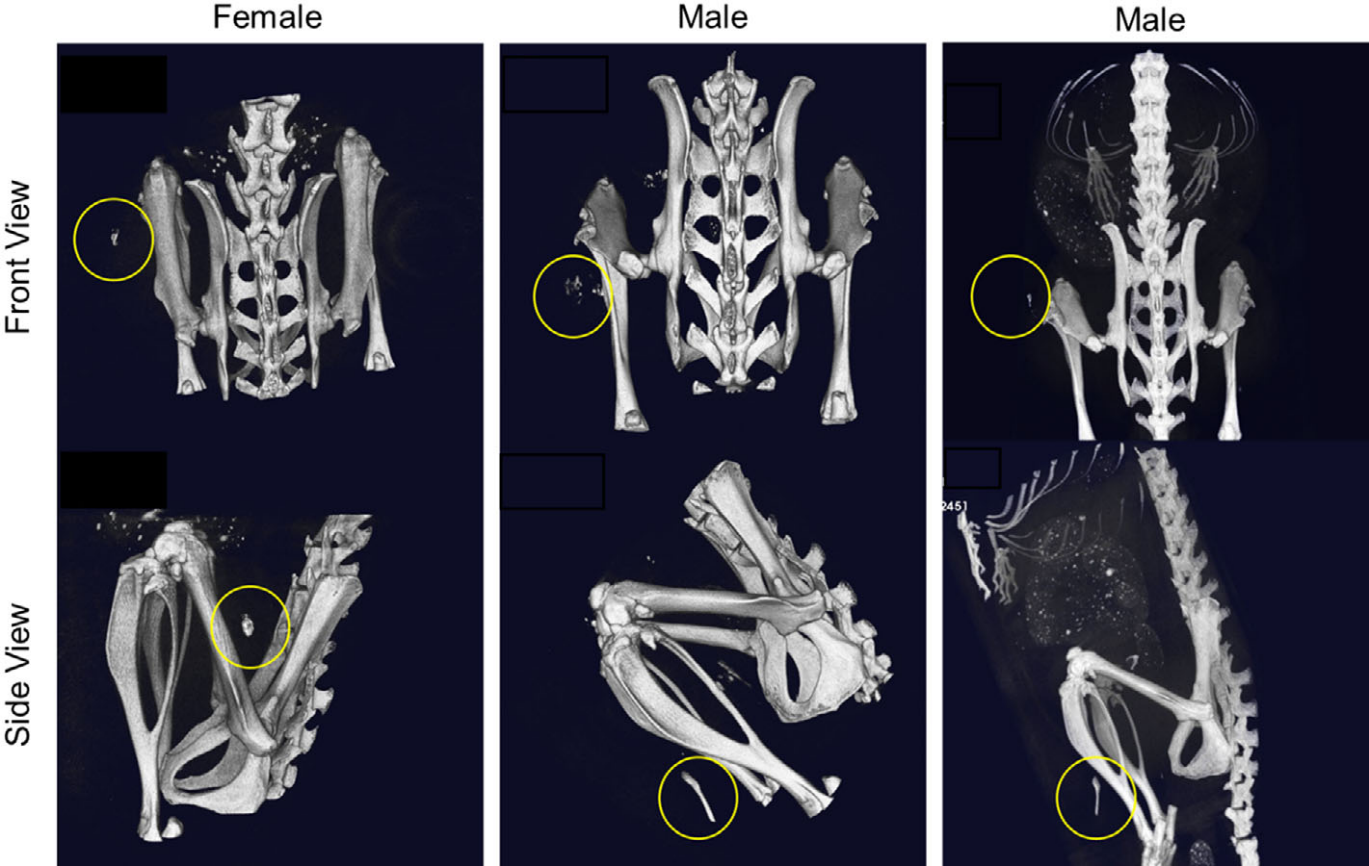
MicroCT images of three mice injected with AuPs into the R hind limb postmortem. Saline injections we performed on the L hind limb as a control. AuPs injection sites are highlighted with a yellow circle. All three replicates shown.

Post‐mortem C57BL/6 mice (AUP 2023‐0215) were injected with AuPs at 100 mg/mL in a 100 μL injection and imaged via MicroCT. The scans revealed the location of the AuPs injection as expected (Figure 6), with detectable threshold values between 337 and 863 Hounsfield Units (HU), with lower values occurring when the injection diffused through the muscle, as expected (Figure S6).

## 3 DISCUSSION

GBM is an aggressive and highly malignant brain cancer with poor prognoses and high mortality rates. It is crucial that this cancer is discovered early for treatments to be viable and successful for patients. MRIs are the most widely accepted and used imaging modality for GBM due to their higher tumor imaging sensitivity.^26^ Although a valuable tool, MRIs are not readily accessible for a large portion of patients, only being present in 66% of all major care facilities.^3^ CT imaging is more cost‐effective, easily accessible, and provides results faster than MRI, resulting in more efficient assessment. Yet, CT contrast agents cause major negative outcomes in patients, including nausea, changes in blood pressure, dizziness, and fevers.^28^ Here, we combined the benefits of AuNPs as CT contrast agents^26^, ^27^, ^38^ with the protection provided by the bi‐layer membrane and the ability to hold high concentrations of AuNPs provided by the large volume internal vesicle core of the polymersome^28^, ^39^ to develop AuPs that lead to superior CT contrast enhancement that could be modified for on target delivery and imaging of glioblastoma.

AuPs are formed at a small overall average diameter <150 nm, which is similar to the size of other polymersomes formed using the same PEG(1000)‐b‐PLA(5000) block copolymer.^32^, ^33^, ^40^, ^41^ TEM images (Figure 2b) are in good alignment with the DLS data (Table 1), although slightly smaller in estimated diameter, likely due to the inability to visualize the exterior PEG layer which can be measured via hydrodynamic diameter in DLS. The use of lyoprotectant mannitol can help maintain the polymersome characteristics during freeze drying, which is required for TEM analysis, although it can lead to slight shrinkage.^42^ This smaller size can be beneficial for the delivery to tumors like GBM which experience the enhanced permeation and retention effect.^43^, ^44^

Most shockingly, AuPs are loaded with almost 0.1 mg of AuNPs per mg of polymer, visualized by complete saturation of the polymersome structure (Figure 2c). In a pentablock terpolymer polymersome made from polycaprolactone, polyethylene oxide, and poly(2‐vinylpyridine), Popescu and Tsitsilianis observed much more punctate AuNPs visible via TEM. Despite their much smaller size (~24 nm), these polymersomes were not saturated with AuNPs like our larger system.^45^ Liao et al. had a similar finding, with PEG‐polycaprolactone polymersomes of ~175 nm in diameter, loading seemingly only individual gold nanorods.^46^ He et al. also observed much less diffuse loading of AuNPs into their ~polycaprolactone‐polyethylene oxide polymersomes that were ~211 nm in diameter, although their AuNPs were modified with cancer therapeutic doxorubicin. Salzer et al. incorporated AuNPs into the hydrophobic bilayer of polymersomes, which has a much smaller volume than the core, and ended up almost completely saturating the membrane at AuNP concentrations of 0.140%w/v.^47^ Most other researchers have not quantified the loaded content of AuNPs into polymersomes. However, DiazDuarte‐Rodriguez et al. did calculate that between 0.03 and 0.04 mg of gold nanorods were loaded per mg of PEG‐b‐poly(N,N‐diethylaminoethyl methacrylate) polymersomes with diameters around 100 nm. Our loaded content was near 0.1 mg AuNP/mg polymersome, which is between 2.5 and 3 fold greater. We hypothesize that this increased loading content has to do with our specific polymer properties and the introduction of a heating step in our loading protocol. PLA is a glassy polymer, which has a neat glass transition temperature (T_g_) between 50°C and 60°C depending on its molecular weight. However, the addition of PEG decreases the observed T_g_.^48^ Specifically, PEG(2000)‐b‐PLA(5000) had a measured T_g_ of 23°C.^49^ By heating our polymersomes to 37°C, we likely made the PLA membrane more fluid to AuNPs, allowing more to travel across the membrane and into the vesicle core. Cooling the AuPs back down to room temperature likely made the PLA membrane glassy again, trapping the encapsulated AuNPs.

AuPs were observed to be biocompatible when incubated with U87‐MG cells (Figures 4 and S3). However, our MTS studies showed that AuNPs caused a linear increase in viability with increasing concentration. This phenomenon has been previously noted in literature and is attributed to the excitation wavelength of the AuNPs interfering with the MTS reagent absorbance.^50^ However, as expected, the same effect is not observed when assessing AuPs, also suggesting the encapsulation of AuNPs. Annexin V/PI studies provided further clarity, demonstrating that AuPs may potentially shield cells from any early AuNP‐induced toxicities, with the highest concentration of AuPs leading to the greatest number of viable cells. This is similar to what was observed in Al Zaki, et al. where the introduction of 5 nm AuNPs into polymeric micelles improved the excretion profile of AuNPs and limited toxicity.^51^ PEG‐b‐PLA polymersomes have been observed to be nontoxic elsewhere.^33^, ^41^ He et al. also demonstrated that their gold‐loaded polymersomes made from polycaprolactone and polyethylene oxide were nontoxic in U87‐MG cells.^52^ AuPs demonstrate concentration‐driven uptake behavior, with 100 mg/mL AuPs leading to enhanced AuNP delivery in comparison to 1 mg/mL free AuNPs (Figure 5). While AuNPs are more readily taken up in GBM cells than most doses of AuPs, these 5 nm particles are unlikely to make it to tumors *in vivo* without direct tumor injection, as their small size makes them likely to be renally cleared upon systemic injection.^53^ PEG‐b‐PLA polymersomes can facilitate enhanced *in vivo* circulation, while still leading to cellular uptake.^33^, ^54^, ^55^ It is important to note that AuNP toxicity is still debated, requiring in‐depth, disease‐specific *in vivo* studies. However, it is suggested that the synthesis method can improve biocompatibility.^56^ Although AuPs enhance contrast at 100 mg/mL, with 10 mg/mL of AuNPs, this is not required for enhanced CT contrast based on both our microCT (Figure 3) and clinical CT (Figure S1) results, indicating that lower doses may be used to decrease the metal load.

AuPs also behave as expected regarding their contrast enhancement in microCT (Figure 3). AuPs at 100 mg/mL carry approximately 10 mg of AuNPs/mL, which is much greater than the 1 mg/mL AuNP standard and thus leads to greater contrast. AuPs maintain their contrast enhancement after *in vivo* intramuscular injections (Figure 6), demonstrating their potential for use in GBM targeting after the addition of BBB‐bypassing targeting ligands like angiopep‐2, Apolipoprotein E, transferrin, or rabies virus glycoprotein.^57^ Most other studies using gold‐loaded polymersomes do not perform CT imaging, instead relying on near‐infrared dye tagging to track particles^46^, ^52^ or ending at *in vitro* assessment.^45^, ^58^ However, Hasannia et al. were able to quantify the contrast enhancement in Hounsfield units after injection of gold nanorod‐loaded peptosomes, similar to polymersomes but also involving peptides. 6 and 24 h post injection directly into tumorized BALB/c mice, they saw a ~1.25‐fold and ~1.4 fold increase in HU compared to PBS injected tumors using microCT.^59^ While this was also calculated using 3D slicer and involved direct injections, the in vivo protocol was significantly different than ours, where we imaged immediately following injection, and the machine used was a clinical CT compared to our assessment using microCT. Similarly, Al Zaki et al. saw that their AuNP‐loaded polymeric micelles, made from PEG‐polycaprolactone, accumulated into a subcutaneous tumor site with visible increases in observed CT contrast.^60^ Notably, these gold nanoparticles also exhibited a radiosensitization effect, acting as a treatment for these tumors. Similarly, Prasad et al. delivered AuNPs using liposomal formulations coated in folic acid to target tumors that were therapeutic when triggered by near‐infrared light which induced heat and reactive oxygen species.^61^ However, their biodistribution was widespread, likely due to the leakiness of liposomes which can be improved by the decreased lateral motility imparted by polymersomes used in this study.^28^ The same group did see improved tumor targeting when developing nanoparticles from cancer‐cell‐derived membrane nanovesicles, but had difficulty delineating the different concentrations of gold nanorods using CT imaging, while these AuPs have distinguishable contrast enhancement with changing concentrations.^62^ AuPs can deliver up to 10 mg/mL of AuNPs, which is well over the reported 0.1 mg Au/mL loaded, suggested to be required for a radiosensitization effect.^60^ This suggests that if AuPs can be targeted to specific tumor sites, they may also act as a simultaneous therapeutic agent.

## 4 MATERIALS AND METHODS

### 4.1 Synthesis and characterization of PEG–PLA/MAL‐PEG‐PLA polymersomes

A 50:50 ratio of polyethylene glycol (1000 Da)‐b‐polylactic acid (5000 Da) (PEG‐b‐PLA) (Polysciences, Inc., Warrington, PA, USA) to maleimide‐functionalized PEG–PLA polymers (MAL‐PEG‐b‐PLA) (Creative PEGWorks, Durham, NC, USA) was dissolved into a 0.1% dimethyl sulfoxide (DMSO, Sigma‐Aldrich, St. Louis, MO, USA) solution to synthesize polymersomes. The organic polymer solution was injected into an aqueous 2 wt% mannitol (Sigma‐Aldrich, St. Louis, MO, USA) solution at a rate of 5 μL/min. Then, 0.002 μL/mL of cystine modified trans‐activator transcription peptide (TAT, Genscript, Piscataway, NJ, USA) was added to the solution and stirred for 30 min post‐injection. The diameter, polydispersity index (PDI), and zeta potential were analyzed before and after the addition of TAT by dynamic light scattering (DLS) using a Malvern Zetasizer Nano (Malvern Panalytical, Malvern, Worcestershire, UK). Polymersomes with sizes between 100 nm and 200 nm with zeta potentials in the range of 20–30 mV were selected for use in in vitro studies.^63^ Polymersomes were stored at −20°C before being freeze‐dried at −105°C and 0.004 MPa using a 4.5‐L capacity benchtop Labconco FreeZone Benchtop Freeze Dryer (Labconco Corporation, Kansas City, MO, USA).

### 4.2 Characterization and clinical CT imaging and analysis of gold nanoparticles

Sterile gold nanoparticles (5 nm diameter; nanoComposix, San Diego, CA, USA) were suspended in various concentrations of PBS to make dilute concentrations of AuNP samples with concentrations of 0, 0.05, 0.1, 0.2, 0.4, 0.8, and 1 mg/mL. With a three‐dimensional computer‐aided design program, SOLIDWORKS, an imageable tray for computed tomography (CT) imaging was printed. The AuNP samples were placed in Eppendorf tubes and held in a three‐dimensional printed imaging tray. The samples were imaged using an Aquilion TSX‐101A CT scanner (Toshiba, Tokyo, Japan). After imaging, DICOM files were exported for further analysis. Horos, a medical image viewer, was used to analyze the images and determine the X‐ray attenuation values of each sample.

### 4.3 Preparation of gold nanoparticle‐loaded polymersomes

Sterile Texas red fluorophore‐labeled gold nanoparticles (5 nm diameter; Nanopartz, Loveland, CO, USA) or sterile gold nanoparticles (5 nm diameter; nanoComposix, San Diego, CA, USA) were loaded into the polymersomes using standard protocols.^33^, ^64^, ^65^ One milliliter of stock gold solution at a concentration of 1 mg/mL and 100 mg of polymersomes were combined and heated using a Corning LSE digital dry bath (Corning Inc., Corning, NY, USA) for 1 h at 37°C. Polymersomes were then cooled for 1 h at 4°C and sonicated at 20% output amplitude for 30 min. The polymersomes were then filtered with Amicon Ultra‐0.5 centrifugal filter devices (Millipore Sigma, St. Louis, MO, USA) in an Eppendorf Centrifuge 5424 R (Eppendorf, Hamburg, Germany) at 14,000 rpm for 15 min at 25°C to remove excess gold. PBS was added to wash the polymersomes to get rid of any adsorbed excess gold, which was centrifuged again at 14,000 rpm for 15 min at 25°C for removal. Filters were then flipped and spun at 14,000 rpm for 2 min at 25°C to collect polymersomes that were then filtered using a 0.45‐μm sterile syringe filter (VWR, Radnor, PA, USA).

### 4.4 Transmission electron microscopy (TEM) imaging

Transmission electron microscopy (TEM) images were obtained using an H9500 TEM machine (Hitachi, Tokyo, Japan). The unloaded TAT‐labeled polymersomes were stained with uranyl acetate as a contrast agent. The TAT‐labeled polymersomes loaded with 1 mg/mL AuNPs were imaged without staining.

### 4.5 Encapsulation efficiency and loaded content of AuNPs in AuPs

AuPs were centrifuged via Amicon Ultra‐0.5 centrifugal filter device (100 kD) in an Eppendorf Centrifuge 5424 R (Hamburg, DEU) at 14,000 rpm for 15 min three times to remove excess gold. Washes were collected to calculate unloaded AuNP concentration and corresponding loaded content and encapsulation efficiency (565 nm via UV/Vis in a Biotek Synergy H1M plate reader) using the following equations.

(1) $$\begin{array}{l} \text{Loaded Content of AuNPs in AuPs}\\ \;=\frac{\text{Total Mass of AuNP}-\text{Filtered Mass of AuNP}}{\text{Mass of Polymersomes}} \end{array}$$

(2) $$\text{Encapsulation Efficiency}=\left(\frac{\text{Loaded mass of AuNP}}{\text{Added mass of AuNP}}\right)\cdot 100$$

### 4.6 Cell culture

U87‐MG cells (ATCC, Manassas, VA, USA) were grown in Eagle's Minimal Essential Medium (EMEM) (Thermofisher, Waltham, MA, USA), supplemented with 10% fetal bovine serum (FBS) (Thermofisher, Waltham, MA, USA), and 1% penicillin (P/S) (Thermofisher, Waltham, MA, USA). The cells were incubated at 37°C in 5% CO_2_.

### 4.7 Cell viability

For MTS, U87‐MG cells were seeded in 96‐well microtiter plates at a density of 5000 cells/well and incubated at 37°C and 5% CO_2_ for 24 h. Cells were then treated with various concentrations of AuNPs and AuPs and incubated at 37°C and 5% CO_2_. After 48 h of treatment, 20 μL of MTS reagent was added to each well and left to incubate for another 3 h. The absorbance of each plate was measured using a Synergy LX multimedia reader (BioTek Instruments Inc., Winooski, VT, USA). For Annexin V/PI, U87‐MG cells were seeded in a 96‐well microtiter plate at a density of 5000 cells/well and incubated at 37°C and 5% CO_2_ for 24 h. Cells were then treated with various concentrations of AuNPs and AuPs and incubated at 37°C and 5% CO_2_. After 24 h, the treatments were removed and replaced with fresh 10% FBS transfection media. Forty‐eight hours after treatment, the cells were washed with PBS and then isolated. The eBioscience Annexin V‐FITC kit (Thermo Fisher Scientific, Waltham, MA) was used to perform Annexin V/Propidium Iodide staining according to the manufacturer's protocol. Briefly, all samples were resuspended in 100 μL of binding buffer (unstained) or 95 μL of binding buffer and 5 μL of fluorochrome‐conjugated Annexin V (stained groups only) and incubated covered for 15 min at room temperature. Cells were then centrifuged and washed in 100 μL of binding buffer. To resuspend cells, 200 μL of binding buffer (unstained) or 195 μL of binding buffer and 5 μL of propidium iodide (stained) were added. All samples were analyzed using the Attune NXT acoustic flow cytometer (ThermoFisher Scientific) with the BL1 and YL2 lasers. Data analysis was conducted using FlowJo™ software (BD Life Sciences) to divide the population into quadrants. Gating was performed against unstained cells to ensure the cell population was within the lower left quadrant, indicating no staining.

### 4.8 Assessment of AuNP and AuP uptake in GBM cells

U87‐MG cells were seeded in 12‐well microtiter plates at a density of 15,000 cells/well and incubated at 37°C and 5% CO_2_ for 24 h. The cells were then treated with various concentrations of AuPs, ranging from 0 to 100 mg/mL, with a positive control (1 mg/mL fluorescent AuNPs). The treated cells were then incubated for 24 h at 37°C and 5% CO_2_. After 24 h, Hoechst stain was added to each well to stain cellular nuclei. The treated cells were fluorescently imaged at 20× magnification using an EVOS XL Core Fluorescent microscope (Thermo Fisher, Waltham, MA, USA).

For flow cytometry, U87‐MG cells were seeded in 24‐well microtiter plates at a density of 50,000 cells/well and incubated at 37°C and 5% CO_2_ for 24 h. The cells were then treated with various concentrations of AuPs, ranging from 0 to 100 mg/mL, with a positive control (1 mg/mL fluorescent AuNPs). The treated cells were then incubated for 24 h under standard culture conditions. After incubation, the cells were centrifuged in an Eppendorf Centrifuge 5424 R (Hamburg, DEU) at 1500 rpm for 3 min at 25°C and resuspended in 500 μL of PBS. The internalization of the treatments was determined using an Attune NxT Flow Cytometer (Thermo Fisher Scientific) at a wavelength of 615 nm.

### 4.9 Micro‐computed tomography imaging of AuPs

A 2% agarose solution was prepared by combining 0.4 g of agarose and 20 mL of PBS and heating the solution, using a microwave, at 15 s intervals until the solution began to boil. The solution was then cooled on benchtop to 37°C. After cooling, 250 μL of the 2% agarose solution was added to 250 μL of AuPs, with concentrations ranging from 0 to 100 mg/mL, as well as a positive control (1 mg/mL fluorescent AuNPs), and individual solutions were placed into Eppendorf tubes. The solutions were then triturated to ensure a homogeneous solution and left benchtop for further settling. Once prepared, individual Eppendorf tubes were imaged using a Bruker SkyScan 1176 Micro CT machine (Micro Photonics Inc., Allentown, PA, USA).

### 4.10 In vivo imaging assessment

Animal Ethics Statement. Animal Use Protocol (AUP) 2023‐0215 was approved by Clemson University's Institutional Care and Use Committee (IACUC), which confirmed that all animal experiments were in accordance with the National Guide for the Care and Use of Laboratory Animals.

Three C57BL/6 mice (1 female, 2 male) were injected intramuscularly immediately postmortem with AuPs in the right hind limb and volume‐matched PBS in the left hind limb (Clemson University AUP 2023‐0215). AuPs were injected at a concentration of 100 mg/mL in 100 μL. Mice were then imaged in a Bruker SkyScan 1176 Micro CT machine with ring reduction (90 kV, 88 μA, 36 FOV, 4 min scan time). Micro‐CT images were analyzed using 3D Slicer 5.8.1. Individual DICOM files were uploaded as volume renderings. Using the “Segment Editor” module, threshold values were applied based on voxel intensities to isolate specific tissue types and materials of interest. X‐ray attenuation values, represented in Hounsfield units (HU), were determined by applying intensity‐based thresholding to observe isolation and visibility of specific regions of interest, with a focus on detection of AuPs.

### 4.11 Statistical analysis

All data was calculated with ± SEM and analyzed via Brown‐Forsythe and Welch One‐Way ANOVA testing with Dunnett T3 multiple comparison tests using GraphPad Prism.

## 5 CONCLUSIONS

This study aimed to assess the ability of AuPs to increase the contrast of CT images with the goal of increasing accessibility to high quality diagnosis of tumors and an initial focus on GBM. The physicochemical characteristics of AuPs could aid in the efficient delivery of treatments to tumor sites, demonstrated by their small diameters, high polydispersity, and high AuNP carrying capacity. The loading capability of the polymersomes was almost 100% across all concentrations and significantly greater than the AuNP‐loaded content observed in the current literature. The modified T_g_ of PEG‐b‐PLA likely enabled this high load. AuNPs revealed a linear increase in contrast related to concentration, both visually and quantitatively, confirming that AuNPs could work well as CT contrast agents. AuPs at concentrations of up to 100 mg/mL were cytocompatible with a glioblastoma cell line (U87‐MG), suggesting that the delivery system did not elicit an unwanted immune response. Increasing concentrations of AuPs revealed an increased internalization into a glioblastoma cell line; although the free AuNPs were also taken up into U87‐MG cells, this is unlikely to translate to in vivo on‐target delivery due to their small size. The high concentrations of AuPs also exhibited increased contrast enhancement in phantom microCT assessment compared to free AuNPs. Finally, AuPs maintain their detectability via microCT upon injection in C57BL/6 mice, with HU between 337 and 863. Findings are encouraging towards the development of a tumor targeting contrast agent for CT imaging. The ease of ligand attachment on the polymersome surface and the successful use of various ligands for GBM targeting is encouraging,^66^ but would need to be validated in future studies using relevant animal models to confirm the translational impact of the technology. In summary, AuPs show promise for high AuNP loading and delivery as a CT contrast agent and could expand the usability of CT for tumor imaging applications. Future studies will need to focus on elucidating the pharmacokinetic and pharmacodynamic behavior of AuPs in an orthotopic GBM model.

## AUTHOR CONTRIBUTIONS

**Emily Barnett**: Investigation, writing—original draft, data curation, methodology, validation, formal analysis. **Joey Lavalla**: Investigation, writing—original draft, methodology, validation, data curation, formal analysis. **Pranavi Thatavarthi**: Investigation, methodology, validation, data curation, writing—review and editing. **Isabel Ray**: Investigation, data curation. **Taylor Hamas**: Investigation, data curation. **Jessica Jager**: Investigation, data curation. **Vaishnavi Kanduri**: Investigation, data curation. **Jasmine White**: Investigation, data curation. **Elizabeth Singleton**: Investigation, data curation. **Jordan Drinks**: Investigation, data curation. **Megan Pitz**: Investigation, methodology, validation, data curation. **Angela Alexander‐Bryant**: Conceptualization, investigation, funding acquisition, writing—review and editing, supervision, project administration. **Jessica Larsen**: Conceptualization, investigation, funding acquisition, writing—original draft, writing—review and editing, validation, visualization, supervision, resources, project administration.

## FUNDING INFORMATION

This work was supported by Clemson's Creative Inquiry Program and internal research grants. Dr. Jessica Larsen was supported by the National Institutes of Health National Institute of Neurological Disorders and Stroke (R21NS133477, DP2NS148060, and R01NS142877) and National Institute of General Medical Sciences (P20GM146584 and P20GM103499) throughout the duration of this work.

## CONFLICT OF INTEREST STATEMENT

The authors declare no conflicts of interest.

## Supporting information


**Data S1:** Supporting Information.

## Data Availability

The data that support the findings of this study are available from the corresponding author upon reasonable request.
