# Supplementary material for: Enabling in vivo imaging in low‐resource settings: Computed tomography imaging of gold‐loaded polymersomes for the detection of glioblastoma
Source: Bioeng Transl Med. 2026 Jan 27;11(3):e70109. doi: 10.1002/btm2.70109 (PMC13247402; doi:10.1002/btm2.70109)
Supplement: Supplementary file 1 — Data S1: Supporting Information. [file BTM2-11-e70109-s001.docx]

**Supporting Information**

**Enabling *in vivo* imaging in low-resource settings: Computed tomography imaging of gold-loaded polymersomes for the detection of glioblastoma**

Emily Barnett^1^^, Joey Lavalla^1^^, Pranavi Thatavarthi^2^, Isabel Ray^1^, Taylor Hamas^1^, Jessica Jager^2^, Vaishnavi Kanduri^1^, Jasmine White^1^, Elizabeth Singleton^2^, Jordan Drinks^1^, Megan Pitz^1^, Angela Alexander-Bryant^1^, Jessica Larsen^1,2*^

^1^Department of Bioengineering, Clemson University, Clemson, SC, USA
^2^Department of Chemical and Biomolecular Engineering, Clemson University, Clemson, SC, USA
^^^co-first author

***Corresponding Author:**
Jessica Larsen
130 Earle Hall, Clemson, SC 29634
[larsenj@clemson.edu](mailto:larsenj@clemson.edu)


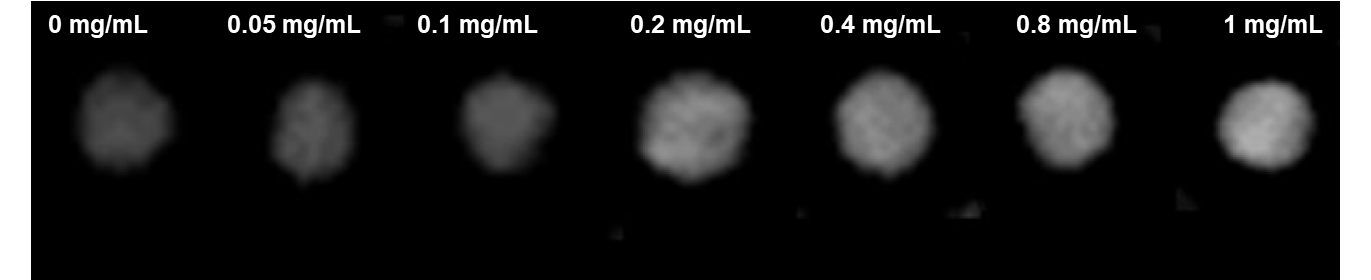


Supplemental Figure 1. Top-down images of AuNPs at various concentrations using Clinical CT. These images enabled the calculation of X-ray attenuation via Horos software.


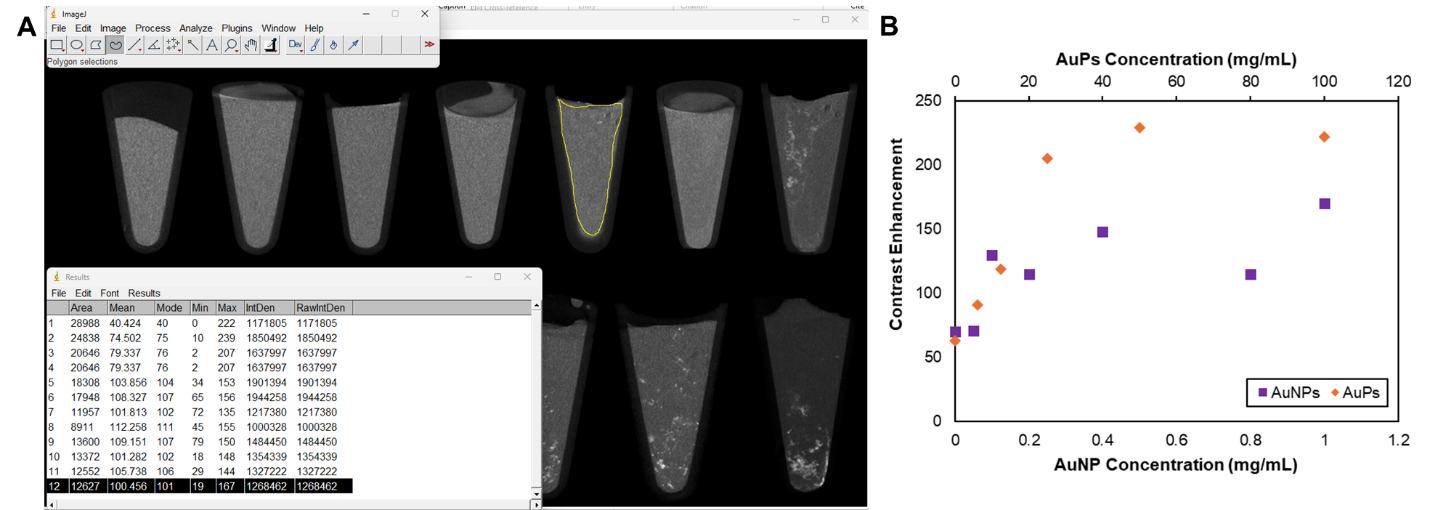


Supplemental Figure 2. A. Image J quantification of pixel intensities in each microCT phantom image. Region of Interest (ROI) analysis was performed to quantify the minimum and maximum gray values within. Maximum minus minimum gray values allowed us to quantify contrast enhancement. B. Contrast enhancement calculated via ImageJ versus AuNP and AuP concentration.


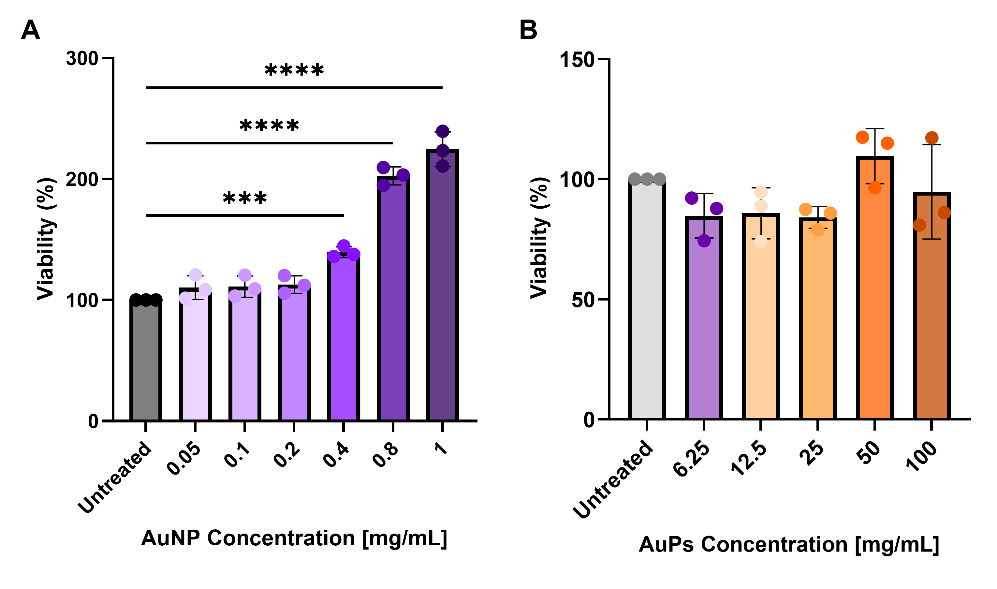


Supplemental Figure 3. MTS Proliferation Assay performed on U87-MG cells to determine (A) 5 nm sterile gold and (B) gold-loaded TAT-labeled polymersomes on viability. n=3; ***p <0.001; **** p< 0.0001


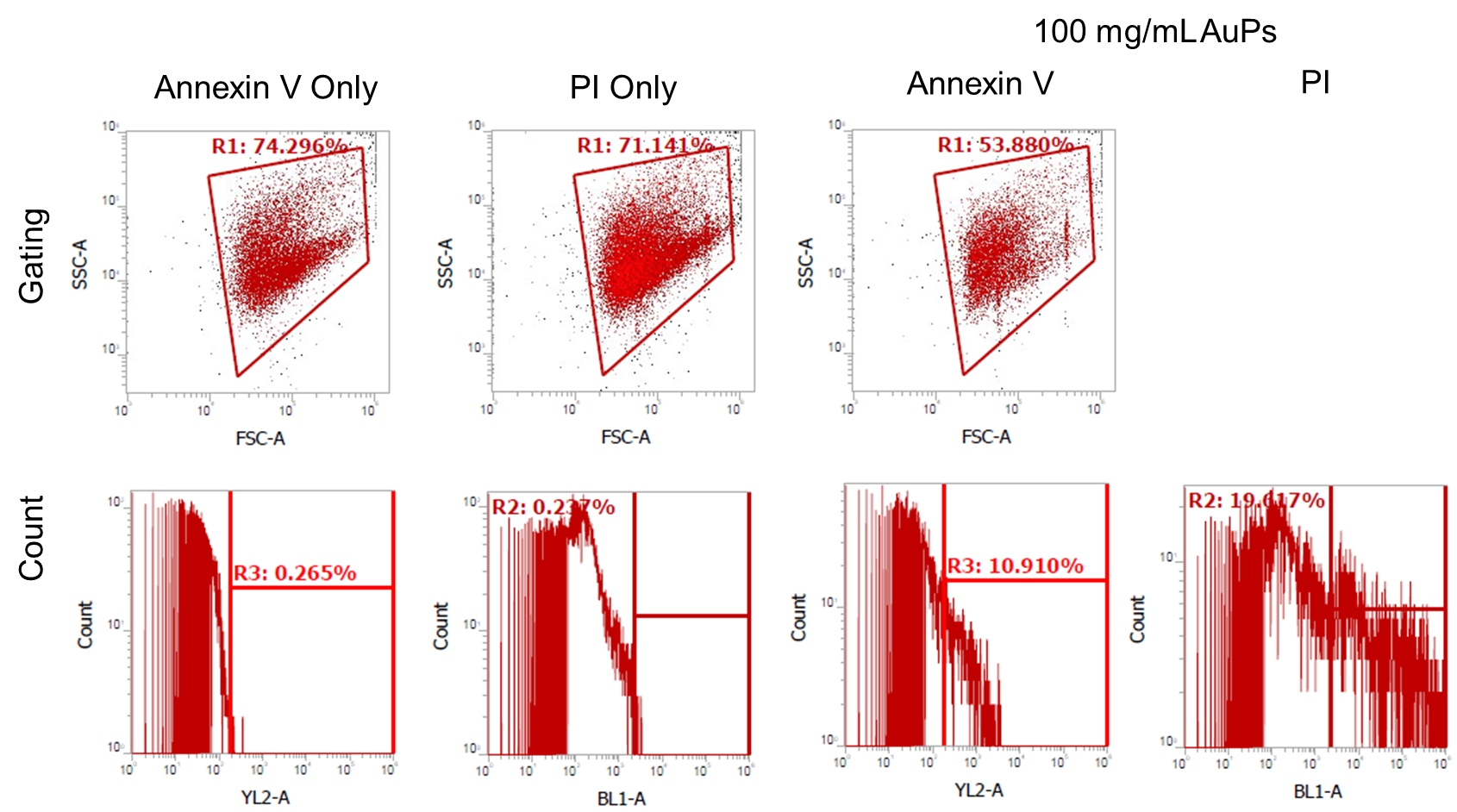
*Supplemental Figure 4. Flow cytometry gating used for Annexin V/PI staining. Example data from Annexin V only and PI only controls, followed by one of the replicate experiments using 100 mg/mL AuPs.*

Supplemental Figure 5. Flow cytometry gating used for uptake analysis. Example data from (A) Untreated U87-MGs, (B) 100 mg/mL AuPs treated U87-MGs


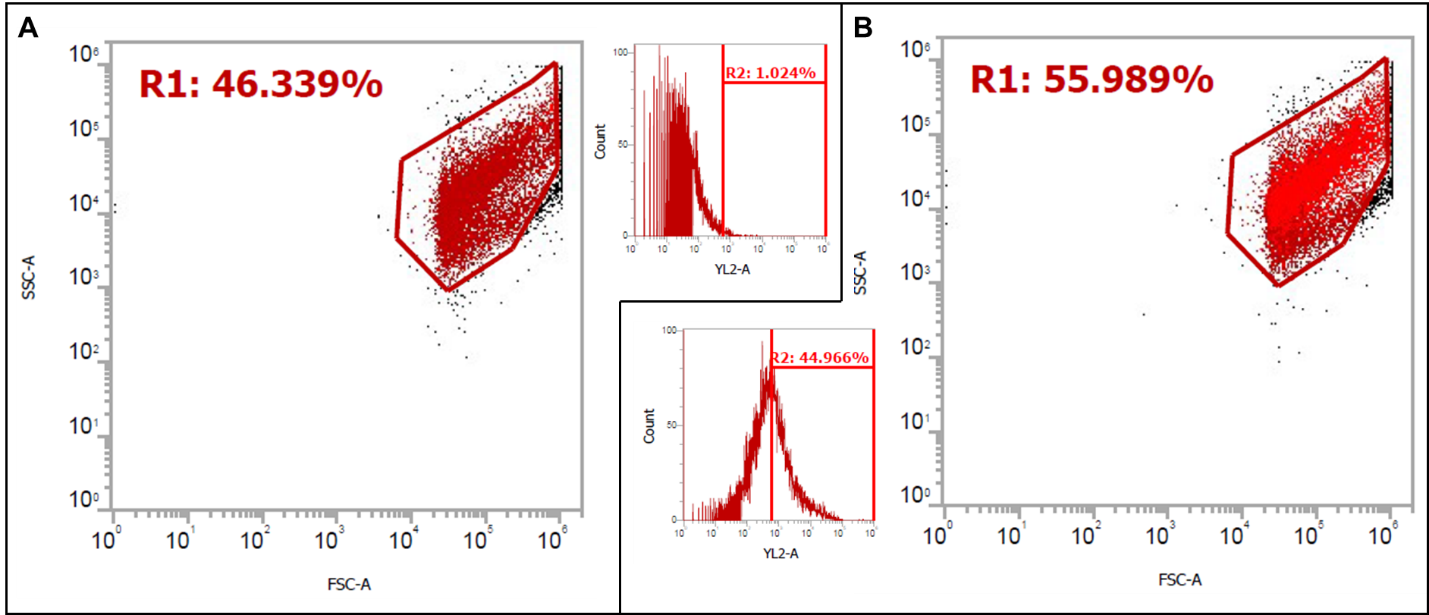

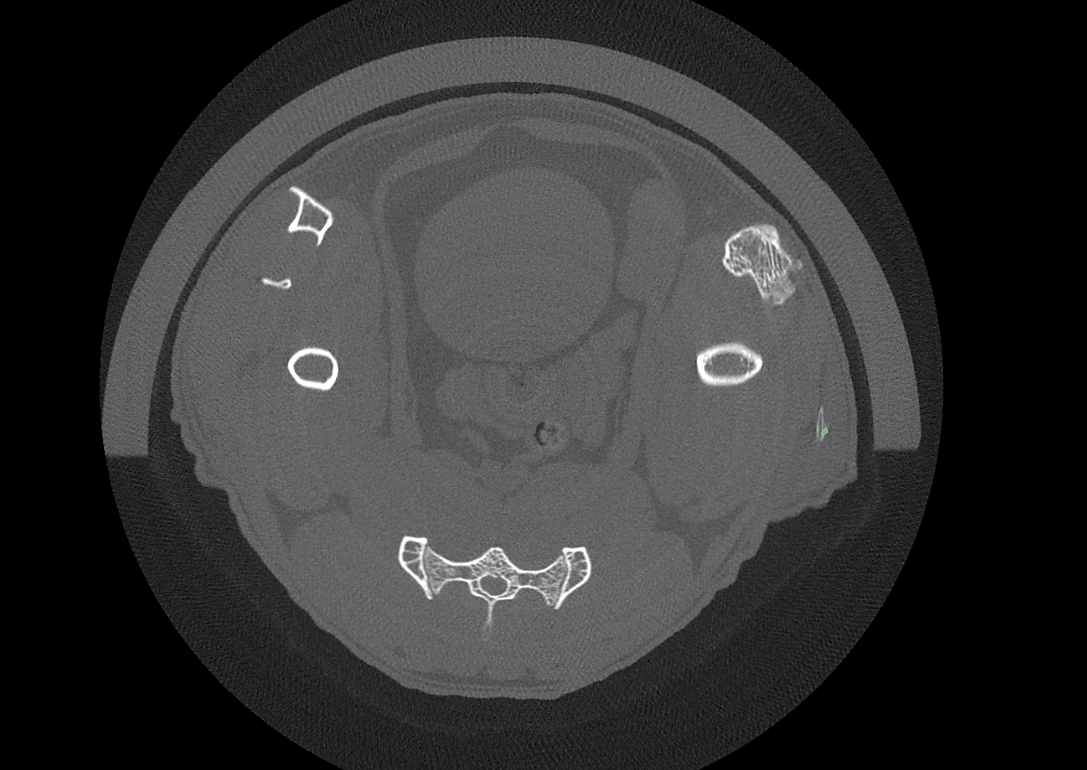

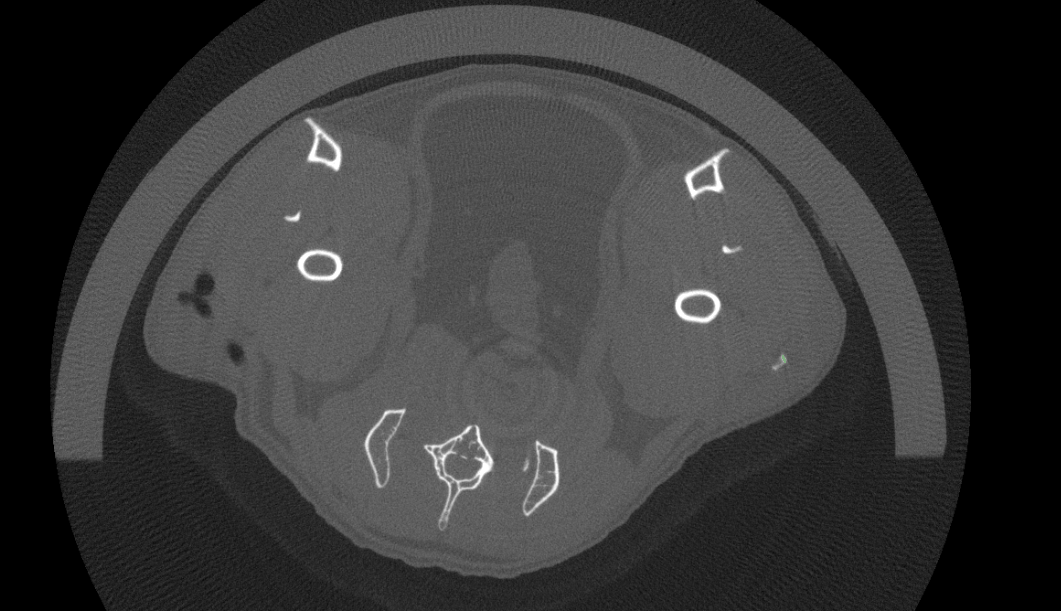


Supplemental Figure 6. Example 3D Slicer Analysis, with AuPs highlighted in green, corresponding to set HU
